# The Necessity and Feasibility of Implementing Regenerative Practices in Pasture-Based Beef Cattle Farming

**DOI:** 10.3390/ani16152384

**Published:** 2026-08-03

**Authors:** Ferenc Szabó, Gabriella Holló

**Affiliations:** 1 Department of Animal Sciences, Albert Kázmér Faculty, Széchenyi István University, H-9200 Mosonmagyaróvár, Hungary; szabo.ferenc@sze.hu; 2 Institute of Animal Sciences, Kaposvár Campus, Hungarian University of Agriculture and Life Sciences, H-7400 Kaposvár, Hungary

**Keywords:** regenerative agriculture, pasture based, cattle farming, grazing methods

## Abstract

This review analyzes sustainable and regenerative development in the beef production chain, focusing on environmental impacts and pasture-based cattle farming. Livestock emissions account for about 14.5% of global anthropogenic greenhouse gases, with beef cattle contributing 3–3.2% mainly from enteric fermentation and manure management. Pasture-based cow-calf operations generate 60–70% of these emissions, posing significant challenges for producers. Improper grazing degrades soil and vegetation, reducing productivity, while climate change and technology introduce new animal health issues. At the same time, given that beef is an important food item worldwide, it is important to strive for the regenerative management of beef cattle farming. This article lists possibilities for researchers, beef cattle breeders, and farmers to follow and implement, in the hope that the sector will become regenerative. Scientific innovation in grazing-based beef cattle systems should focus on precision livestock monitoring, regenerative pasture management, genetic selection, and improving animal health, as well as identifying opportunities within the value chain. Such innovations could help to optimize productivity, enhance sustainability, improve animal welfare, and generate new income streams through ecosystem services or premium grass-fed markets.

## Simple Summary

Regenerative farming is an approach that restores soil health, increases biodiversity, and improves ecosystem resilience. It reduces the environmental impact by working with natural processes instead of depleting them. Although beef production emits much fewer greenhouse gases than urbanization, industry, and other agricultural sectors, beef farmers still need to consider regenerative development for the future. This review paper draws attention to the importance and possibility of implementing regenerative methods in the pasture-based cow-calf sector of beef production by analyzing literature sources. A detailed description of the sectors of the beef production chain, focusing on the cow-calf operation and grazing systems, highlights the benefits that can be realized through regenerative practices. However, the emerging regenerative cow-calf production paradigm is very promising but still requires comprehensive local research.

## 1. Introduction

Beef production is an important part of global animal husbandry, converting nonedible plant materials into a highly nutritious food source. However, it also has an environmental footprint, including greenhouse gas emissions, changes in land use, water consumption, and biodiversity loss. Several studies have been published that quantify these impacts and identify opportunities for mitigation [1,2]. Globally, livestock-related emissions account for around 14–15% of total anthropogenic greenhouse gas emissions, with beef cattle contributing 3.0–3.2% due to enteric fermentation and manure management [3,4]. However, although beef cattle farming is not the largest greenhouse gas emitter, its environmental impact still needs to be addressed. Beef cattle farming is a land-intensive activity based on pasture and feed crops, which can result in deforestation in some regions. Rotz [3] and Pelton et al. [5] emphasize that changes in land use, particularly in regions where grazing is intensive, increase the carbon footprint of beef production. Overall, the literature highlights that beef cattle production poses significant environmental challenges but also suggests ways to mitigate these. According to several specialists, further research is needed to evaluate the global effects on water and biodiversity, GHG emissions, and the socio-economic feasibility of mitigation strategies. Furthermore, cross-regional comparative studies would facilitate the identification of best practices that can be adapted across production systems. The aim of this paper is to review the characteristics and environmental impacts of beef cattle farming based on literary sources, and to attempt to summarize the options with which the adverse impacts of the sector can be mitigated. Nevertheless, we believe that our manuscript presents a new concept and a novel approach. In the fields of animal husbandry and beef cattle farming, both experts and the existing literature have primarily focused on sustainable development, without fully considering the potential of regenerative development. As regenerative development is an emerging approach that also encompasses environmental dimensions, we believe that our paper offers a meaningful contribution and can be regarded as novel.

The aim of our review article is to summarize the latest results and to draw attention to the environmental impacts of pasture-based beef cattle farming and the possibilities of regenerative development alongside sustainability.

In the literature review, we followed the logic of the technological processes of the vertical chain of beef production and grass-based beef cattle farming as a protocol. When searching for literature sources, we initially used the keywords ‘development of the relationship between humans and nature’, ‘regenerative agriculture’, ‘pasture-based cattle farming’, ‘greenhouse gas emissions’, ‘biodiversity’, ‘soil health’, and ‘grazing methods’. Looking through, the sources’ duplicates were removed. We only considered and incorporated data into our manuscript that came from high-quality, peer-reviewed publications and from authoritative international and national regulatory sources (FAO, IPCC, USDA, etc.).

**Figure 1:**
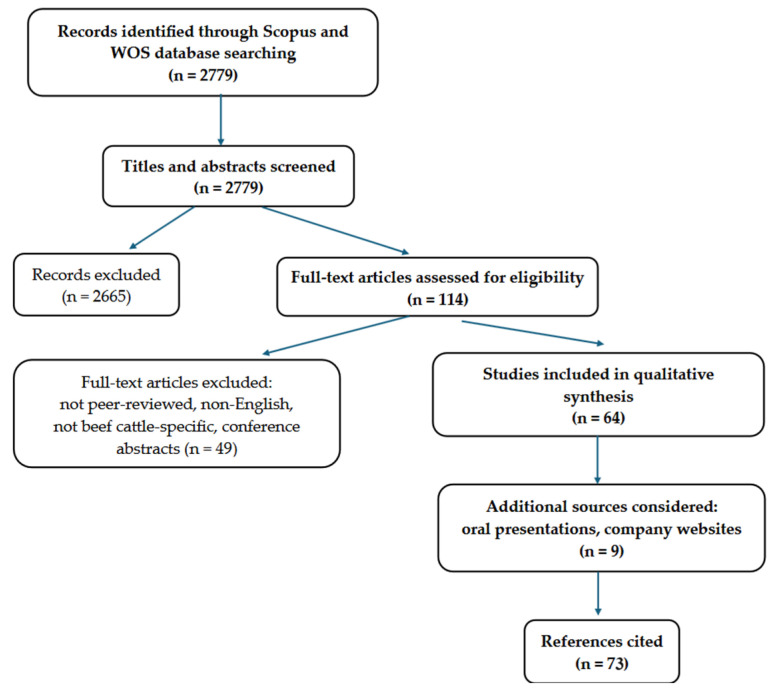
Flow diagram of the study selection process.

The initial search identified 2779 records from Scopus and Web of Science for the period of 1990–2025 relating to analyses of grass–livestock interactions in relation to cattle production systems. Of the publications found, only a few that were related to pastoral farming were considered. We then searched for the terms ‘sustainable’ and ‘regenerative farming’. A few articles emerged here too, of which we reviewed those related to the beef cattle sector in detail. We also searched for literature on the vertical sectors of the beef industry and analyzed articles that dealt with the environmental impact, especially greenhouse gas emissions. We then searched for literature on grazing cow-calf production and the use of different grazing methods. Sources that did not include substantive data on environmental impacts were excluded. After filtering, 114 remained for detailed records. Following title and abstract screening, 49 records were excluded for not meeting the inclusion criteria (e.g., not peer-reviewed, not beef, or conference abstracts). Ultimately, 64 studies were included in the qualitative synthesis. In addition, there were 9 relevant and recent supplementary sources (e.g., university, or institution report), as a total of 73 citation were considered (Figure 1).

## 2. Humans and Nature

The relationship between humans and natural resources has changed dramatically throughout history [6,7,8,9].

Since early humans lived in small nomadic groups and did not engage in agricultural, animal production, or industrial activities, their impact on the environment was minimal [10]. When local resources became limited, they simply moved to new areas, rather than overexploiting environmental resources [11]. Their way of life was therefore sustainable.

As human civilization developed, the use of natural resources increased. This led to environmental changes, including pollution, deforestation and urbanization.

Nowadays, human societies use more natural resources than the Earth can replenish each year. This is known as an ecological challenge [12,13,14,15].

According to the IPCC’s Sixth Assessment Report [16], here are the global GHG emissions, in CO_2_-equivalents (CO_2_-eq) to account for the different global warming potentials of various greenhouse gases: energy (electricity and heat production): 34%, industry: 24%, agriculture, forestry, and other land use 22%, transport: 15%, and buildings: 6%.

According to the Food and Agriculture Organization (FAO), livestock supply chains are responsible for approximately 14.5% of global anthropogenic greenhouse gas (GHG) emissions. Within this sector, cattle—encompassing both beef and dairy production—account for about 62% of the total livestock-related emission; of this, beef production contributes around 35% [17]. This means that the beef cattle sector gives 3.15% of total emission.

The expansion of agriculture, deforestation, and urbanization has fragmented habitats and reduced ecosystem resilience. According to Larigauderie et al. [18], transformative systemic changes are urgently needed to halt biodiversity decline, as up to 1 million species are currently at risk of extinction globally.

Kopittke [19] emphasizes that soil degradation threatens agricultural productivity, water retention, and ecosystem health, and has a negative impact on biodiversity and food security.

Together with a growing population and industrialization, the extraction of water, minerals, fossil fuels, and timber is increasing. Xiong and Wang [20] published an article detailing how resource extraction in China has led to severe environmental degradation, including deforestation, groundwater depletion, and pollution, which has impacted sustainability.

Some renewable resources, such as forests and fish stocks, can be replenished if managed sustainably. However, humans replace only a small amount of what we consume [21]. This situation of imbalance has led to climate change, biodiversity loss, soil degradation, and resource depletion.

Today, we are facing the global challenge of finding ways to achieve sustainable development, meeting current needs without compromising the ability of future generations to meet theirs [22]. This involves reducing waste, protecting ecosystems, and shifting towards renewable energy sources. This situation poses a significant global and local challenge for the economy, agriculture, and animal husbandry, including beef cattle farming and beef production.

## 3. Sustainable vs. Regenerative Farming

The modern concept of sustainable livestock farming emerged from the convergence of biological and ethical analyses of livestock systems in the 1990s. Huisman et al. [23] defined sustainability in animal production, establishing that long-term productivity hinges on maintaining soil fertility, feed efficiency, and animal health, rather than maximizing short-term outputs.

Building on this theory, Thompson and Nardone [24] furthered the discussion by placing livestock production within a broader framework of ethical sustainability. They argued that sustainable livestock farming must reconcile environmental integrity, economic viability, and moral responsibility towards animals and rural communities. Their paper introduced the distinction between resource sufficiency and functional integrity approaches, which have since guided sustainability assessments in animal agriculture.

Sustainable agriculture emerged in the mid-20th century as a response to the environmental degradation caused by industrial farming practices. It emphasizes maintaining the health and productivity of farming systems over the long term. The concept gained prominence as concerns about soil degradation, water scarcity, and biodiversity loss became more pronounced [25].

Sustainability in cattle systems spans the environmental, animal welfare, economic, and social dimensions [26]. As Mata [26] mentioned, sustainability for cattle systems were examined with a particular emphasis on greenhouse gas emissions. It also highlighted the integration of animal welfare within One Health, One Welfare, and One Biology perspectives, while identifying persistent methodological gaps in the assessment of long-term performance, welfare indicators, and policy coherence.

The regenerative farming idea later emerged as an extension of the sustainable concept; however, the concept has existed for more than four decades [27]. In the 1980s, Robert Rodale, son of J.I. Rodale, introduced the term “regenerative agriculture” to describe farming practices that not only sustain but also improve and regenerate the health and biodiversity of farming ecosystems [28]. Rodale believed that regenerative agriculture went beyond sustainability by actively restoring soil fertility, enhancing ecosystem services, and improving biodiversity [29]. It has become more common in recent years, especially in the US, South America, Australia, and India, as well as UK [30].

There are key differences between sustainable and regenerative agriculture, animals, and beef cattle farming. Sustainable agriculture focuses on maintaining current environmental conditions to prevent harm [25,31]. This also aims to maintain the current ecological balance by conserving resources, minimizing the environmental impact, and ensuring economic viability. It considers practices such as crop rotation, reduced chemical use, and soil conservation in order to prevent degradation and maintain long-term productivity [25,31].

The concept of regenerative agriculture is to restore and enhance soil health, biodiversity, and ecosystem services. This is often achieved through techniques such as no-till farming, cover cropping, and holistic grazing [28,29]. It emphasizes practices that rebuild soil organic matter, increase biodiversity, and sequester carbon, with the aim of improving the resilience and productivity of agricultural systems [32,33,34]. According to Urdantea [35], sustainable agriculture must consider both plant and animal populations in its management, since they both modify the environment to adapt it to their requirements. Thus, regenerative practices have emerged to restore the self-restoration ability of ecosystems.

Key management practices implemented in cattle farming systems included rotational grazing, the use of multi-species pastures, crop–livestock integration, and biodiversity enhancement through measures such as hedgerow maintenance [27].

## 4. Sectors of Beef Production Chain and Their Ecological Footprint

This section focuses on the ecological footprint of the different sectors of the beef chain and attempts to draw attention to the possibility of change.

### 4.1. Seedstock/Breeding Sector

This sector maintains purebred and composite herds that provide the genetic foundation for the entire beef supply chain. Breeders sell bulls, semen, and replacement heifers selected for their growth rate, feed efficiency, fertility, and carcass quality [36,37]. Genetic progress in this stage strongly influences productivity and environmental performance downstream. The seedstock sector contributes a relatively small share of total emissions of beef production (<5%) since the herd size is limited [38]. Emissions arise mainly from enteric methane (CH_4_) produced by breeding cows and bulls, and from manure management. Genetic improvement in this phase reduces the intensity of lifetime emissions by improving the feed efficiency and reproductive performance [37].

### 4.2. Cow–Calf Operations (Ranch Phase)

Cow–calf producers manage breeding herds to produce calves, which remain with their mothers on pasture until weaning at approximately 6–10 months of age. These operations are the backbone of the beef supply, as they produce feeder calves for the next stages. Management factors such as the reproductive rate, nutrition, and grazing practices strongly affect the system efficiency and greenhouse gas (GHG) intensity [37,38]. The cow–calf phase contributes 60–70% of total GHG emissions across the beef supply chain [38,39]. Major sources include the following: enteric methane from cows and calves, manure decomposition releasing nitrous oxide, land-use change, and soil carbon fluxes. Water and land footprints are also significant—extensive grazing systems dominate the global land use for beef [40]. Mitigation options include improved forage quality, rotational grazing, and feed additives such as 3-NOP or tannins [5].

### 4.3. Stocker/Backgrounding Sector

After weaning, calves may enter a stocker or background phase. During this time, they are either grazed or fed lower-energy diets to increase their frame size prior to entering the feedlot. This stage optimizes the use of forage and grain, reduces the total number of days in the feedlot, and improves the cost efficiency [36,37]. The growth rates and feed conversion efficiency during this phase significantly impact the environmental footprint [5]. This intermediate stage contributes 10–15% of total beef greenhouse gases (GHGs) [37]. The primary impacts are methane from grazing and land occupation. However, well-managed grazing can offset some emissions through soil carbon sequestration [39]. Mitigation strategies include adaptive rotational grazing and improved forage-legume mixes.

### 4.4. Finishing/Feedlot Sector

Feedlots are intensive systems in which cattle are fed high-energy (grain-based) rations in order to reach target slaughter weights and achieve specific carcass compositions. The feedlot sector contributes a similar percentage of total methane emissions as the stocker sector. Feed conversion efficiency, feed formulation, and manure management are critical determinants of profitability and sustainability. Feedlots account for 15–25% of total emissions in grain-fed systems [5,36]. Sources include methane and nitrous oxide from manure handling, CO_2_ from feed production, fertilizer and fuel use, and energy consumption for feed milling and operation. Although feedlots are intensive, they can achieve a lower emission intensity (8–12 kg CO_2_e/kg live weight gain) due to the higher feed conversion efficiency [37].

### 4.5. Processing/Slaughter/Packing

This involves slaughtering, dressing the carcass, cutting it into primal cuts, and packaging it for distribution. Major regulatory and consumer concerns include food safety, humane handling, and traceability [39,41]. By-products such as hides, tallow, and offal are also recovered at this stage, contributing to the total economic value of each animal. This stage accounts for 2–4% of total greenhouse gas (GHG) emissions, primarily due to the electricity usage, refrigeration, and wastewater treatment [39,41]. The water footprint can amount to 400–800 L per kilogram of carcass weight [17]. Improvements in sustainability include energy recovery and water recycling.

### 4.6. Distribution, Retail, and Food Service

After processing, beef enters the cold chain distribution system, ultimately reaching wholesalers, retailers, and the food service sector. Market segmentation and price structures are strongly influenced by branding (e.g., grass-fed, organic, or breed-specific) and consumer demand [17,36]. Supply chain efficiency and product traceability are key research topics at this stage. These stages account for 1–3% of the total greenhouse gas (GHG) footprint [41]. Emissions arise from refrigeration, packaging, and food waste. Globally, retail and consumer waste can add a further 5–10% to the total beef footprint [39]. Mitigation strategies include an efficient cold chain, innovative packaging, and reducing food waste.

### 4.7. By-Products and Non-Food Sectors

Non-meat products, such as leather, bone, and tallow, are used in the production of leather goods, pharmaceuticals and cosmetics. When allocated in life cycle assessments, these co-products can substantially offset the overall environmental footprint [38,40]. These stages account for 1–3% of the total greenhouse gas (GHG) footprint [41].

### 4.8. Support Sectors (Feed, Veterinary Services, Transport, and Finance)

Upstream industries such as feed crop production, veterinary services, transport, and financing underpin all stages of beef production. Feed production alone can account for 45–60% of total supply chain greenhouse gas (GHG) emissions due to fertilizer use, energy consumption, and land-use change [5,39]. Transport contributes a smaller share (less than 2%), but this increases with longer distances between production stages [41]. Mitigation measures include precision agriculture, low-emission fertilizer, and sourcing feed locally.

Looking through the different sectors of the beef production chain, we realize that there are different ecological impacts and some possibilities to embrace more regenerative practices to a certain extent. However, many things need to be clarified regarding improvement.

## 5. Systems for Cow-Calf Operations (Ranch Phase)

However, all of the aforementioned sectors have an environmental footprint. This paper focuses on cow-calf operations because they are part of farming, whereas some of the others are closer to industrial production.

The cow–calf sector is the foundational stage of beef production, in which breeding cows produce and rear calves [36,37]. As previously mentioned, this phase is the most land- and resource-intensive in the beef supply chain [38,39]. This phase establishes the biological and environmental baseline for the entire beef system—animal growth, reproduction rates, and calf survival all depend on the quality and sustainability of the farming system employed. The diversity of cow–calf systems depends on the climate, land availability, and resource inputs.

These systems can be broadly divided into the following categories:

### 5.1. Extensive (Pasture-Based or Rangeland) Systems

This system is dominant globally, especially in North and South America, Australia, and sub-Saharan Africa. Cows and calves graze on native or improved rangelands, often on low-input, large-area farms. Feed is predominantly grasses, and limited supplemental feeding during drought or winter. The advantages of these systems are the low feed cost and ecosystem services (carbon sequestration, and biodiversity). Its challenges are variable forage quality, drought vulnerability, and lower reproductive efficiency. Although there is high land use, there is potential for soil carbon sequestration under sustainable grazing, which reduces the environmental footprint [37,39].

### 5.2. Semi-Intensive or Mixed Systems

Semi-intensive and mixed systems are found in Europe, South America, and Asia where pasture and crop production are integrated. Animals graze for part of the year and receive crop residues, silage, or concentrates in barns or paddocks. Additive feeding is based on mixed forages, crop by-products, and conserved silage. It has many advantages such as better feed control, reduced land requirement, and improved reproduction and growth. However, it has challenges such as higher input costs and nutrient management issues (risk of manure pollution). However, there are challenges, such as higher input costs and nutrient management (risk of manure pollution). It features immoderate GHG emissions and improved productivity per hectare [5].

### 5.3. Intensive (Confinement-Based) Systems

Intensive systems are common in regions with limited grazing land (e.g., East Asia, and parts of Europe). Cows and calves are kept in barns or dry lots, and fed formulated diets or silage. Feed is generally total mixed ration (TMR), silage, and concentrates. However, it has several advantages, including controlled feeding and biosecurity, and a reduced land pressure. But the high costs of the feed and infrastructure, and the increased need for manure management present a challenge. Overall, it can be characterized by higher emissions from feed production and manure, but a smaller land footprint [37].

### 5.4. Smallholder and Agro-Pastoral Systems

Smallholder and agro-pastoral systems are predominant in developing countries (e.g., Africa, South Asia, and Latin America). Herds are multi-purpose (meat, milk, draft power, and manure). The feeding system is based on crop residues, communal grazing, and household by-products. It has a low capital input and offers social and economic resilience. The challenges are limited veterinary care, poor nutrition, and low productivity. There is a high GHG intensity per kg of beef, but low total emissions per farm [38].

## 6. Possible Impacts of Regenerative Aspects of Cow–Calf Systems

Regenerative cow–calf production emphasizes restoring ecosystem function, enhancing carbon sequestration, and improving resource efficiency while maintaining animal welfare and profitability [42,43].

### 6.1. Soil Carbon Sequestration and Health

Well-managed grazing systems can increase soil organic carbon (SOC) by 0.3–1.0 metric tons of carbon per hectare per year [43,44]. Practices such as rotational or adaptive multi-paddock grazing promote root growth, microbial diversity, and water infiltration. Healthy soils can mitigate the effects of drought and reduce erosion [45]. Example: Stanley et al. [43] found that beef production under adaptive grazing in the US upper Midwest achieved net carbon sequestration, offsetting up to 80% of total system emissions.

### 6.2. Vegetation and Biodiversity Enhancement

Regenerative cow–calf systems help to maintain plant species diversity and support wildlife habitats on native rangelands [42,46]. Periodic rest and grazing rotation mimic natural herbivory patterns, allowing grass to recover and seeds to disperse. A diverse plant cover improves pollinator and soil organism populations [47].

### 6.3. The Water Cycle and Nutrient Efficiency

An improved soil structure enhances rainwater infiltration and reduces surface runoff [44]. Distributing manure across pastures recycles nutrients and reduces the dependence on fertilizers. Integrated crop–livestock systems (semi-intensive) close nutrient loops and increase water use efficiency [46].

### 6.4. Methane and Emission Mitigation

Enhanced forage quality and the integration of legumes reduce the intensity of enteric methane [5]. Adaptive grazing improves feed digestibility and animal productivity, thereby reducing emissions per kilogram of beef produced [42]. The combination of regenerative grazing and feed additives (e.g., tannins or 3-NOP) can further reduce the methane output.

### 6.5. Socio-Economic and Resilience Benefits

Regenerative ranches often demonstrate a greater resilience to drought, reduced dependency on external resources, and improved long-term profitability [43,44]. These systems emphasize animal welfare, community engagement, and intergenerational sustainability. Using regenerative methods, smallholder systems can enhance food security and rural livelihoods [46].

### 6.6. Social Norms Attitudes

Adaptive farmers placed more emphasis on environmental impacts, felt more confident about change, and reported better wellbeing through ties to land and animals. Future research should explore social norms in multigenerational farming and support adoption through education, clearer language, and stronger networks across management styles [48]. Further investigation of these outcomes would provide an important evidence base for diverse stakeholder groups, including consumers who may attribute added value to regenerative products and farmers who may assume substantial transition risks in the absence of robust scientific evidence [27].

### 6.7. EU Policy and Regulation

More explicit guidance on regenerative agricultural practices and methodologies is required from both scientific and policy communities to support effective sector-wide transition and to avoid the inconsistent or misleading classification of production systems [30].

## 7. Impact of Different Grazing Practices

As mentioned above, the cow-calf system based on grazing is the cheapest and most profitable method. Many ranchers believe that, if grazing is not possible or is limited, it is not worth dealing with cow-calf production.

Grazing systems influence how livestock utilize forage resources over time and space, affecting pasture productivity, soil health, and ecosystem functioning. The main systems include continuous grazing and rotational grazing, as well as more intensive forms such as multi-paddock grazing, adaptive multi-paddock grazing, and regenerative grazing.

### 7.1. Continuous or Traditional Grazing

The continuous or traditional grazing systems typically involve continuous stocking, whereby livestock graze large pastures for extended periods without rest. This practice often leads to overgrazing, soil compaction, and a decline in plant biodiversity and soil organic matter [49,50]. Such systems primarily focus on short-term productivity, prioritizing animal weight gain or stocking rates over long-term ecosystem function. While it is simple and low-cost, it often leads to uneven forage utilization, the selective overgrazing of preferred plants, soil compaction, and the loss of plant diversity [50,51]. While some studies show an acceptable livestock performance under moderate stocking, ecological outcomes often decline under heavy use.

### 7.2. Rotational Grazing

This system involves dividing pastures into multiple paddocks and periodically moving livestock to allow plants to recover between grazing periods [51]. Compared to continuous systems, rotational management generally improves pasture condition, forage productivity, and water infiltration if timed appropriately [52]. However, results can vary widely depending on rest periods, grazing intensity, and climatic context.

### 7.3. Multi-Paddock (MP) and Adaptive Multi-Paddock (AMP)

Multi-paddock (MP) and adaptive multi-paddock (AMP) are forms of holistic or regenerative grazing. Adaptive multi-paddock grazing is a rotational grazing approach in which pastures are subdivided into numerous paddocks and livestock are moved frequently—often every few days or less—allowing grazed areas sufficient rest before grazing again [53]. Multi-paddock grazing is a more intensive and flexible form of rotational management, characterized by short-duration, high-density grazing, followed by long rest periods. Managers adjust the frequency of paddock rotation and stocking rates according to the forage growth and rainfall [44]. Evidence indicates the potential for improved soil organic carbon and soil regeneration. McDonald et al. [50] and Mosier et al. [54] found that rotational grazing led to enhanced microbial activity and greater ecosystem resilience. However, these benefits depend on the context and require adaptive, skillful management [54]. By mimicking the natural grazing patterns of wild herbivores, regenerative systems encourage the growth of deep-rooted plant species and promote soil carbon sequestration and enhance water infiltration. This leads to improved ecosystem resilience [55]. Multi-paddock and adaptive practices contribute to long-term soil health, biodiversity, and climate mitigation through carbon storage regenerative grazing. However, researchers note that while regenerative grazing can deliver measurable improvements in the soil carbon and vegetation condition, the magnitude and timeframe of the benefits depend on the climate, soil type, and management intensity [56,57,58]. The transition from traditional to regenerative systems may require several years before ecological and economic gains become evident.

There are many benefits to multi-paddock (MP) and adaptive multi-paddock (AMP) grazing systems; nevertheless, farmers and ranchers must consider many limitations. The main considerations are that not all rotational grazing is regenerative and that not all pasture-based systems sequester carbon. The results also depend heavily on management, climate, soil, stocking rate, and the farm’s economic situation.

## 8. Some Research Findings That Could Help with Regenerative Beef Cattle Farming

Numerous publications draw attention to the fact that the possibility of applying regenerative practices depends primarily on climatic, topographic, soil characteristics, and economic, etc. aspects. Nevertheless, among the numerous previous or new research results, certain aspects can be applied in the given circumstances.

### 8.1. Planting Trees on Pastures Has a Beneficial Effect

In tropical production systems and elsewhere, silvopastoral systems help to reduce heat stress and generate microclimates in grazing areas [59]. This is because the temperature under the canopy of trees is between 2 and 9 degrees lower than in cleared paddocks. Among the recognized benefits of this system, there is evidence of increased time for feed consumption and rumination and improved reproductive efficiency, which is more evident in Bos taurus cattle that are less tolerant to heat, with indicators such as the earlier onset of puberty, greater libido, regularity of the estrous cycle, higher conception rates, and less embryo loss [60,61].

### 8.2. No Disadvantage of Turning to Rotation Grazing

According to Rouquette et al. [62], in approximately 30 published experiments comparing continuous vs. rotational stocking, there was no difference in live weight gain per animal between stocking methods.

### 8.3. Positive Effect on Meat Quality

Gordon [63], based on their research, came to the conclusion that the nutritional superiority of beef from grasslands is characterized by a lower intramuscular fat content and higher values of minerals, such as selenium. The complex multifactorial interaction of pastoral systems is also highlighted, impacting the carcass and meat quality. From another work of research, Congreves [64] reported that the regenerative system promoted a healthier lipid profile, characterized by higher levels of polyunsaturated fatty acids and bioactive lipids. These findings provide new insights into how production systems influence the nutritional quality of beef and underscore the potential of regenerative agriculture to enhance human health through dietary intake.

According to Antunes et al. [65], chuck delivered from regenerative farming system had the highest n-3 PUFA content, primarily due to its higher C18:3 n-3 content, resulting in a lower n-6/n-3 ratio (3.95). Beef from regenerative farming showed higher vitamin E and α-tocopherol (0.58 and 0.56 mg/100 g of meat, respectively), unlike meat from the intensive system.

### 8.4. Effect on Pasture Condition

According to Cosgrove et al. [55], ‘regenerative’ (‘adaptive’) grazing practices are being promoted by some pastoral farmers. An on-farm study that commenced in 2022 is testing if a higher instantaneous stocking intensity, but shorter duration of grazing and longer intervals between grazing events, will improve the phosphorus (P) and nitrogen (N) use efficiency, and carbon (C) sequestration in the soil, while maintaining or increasing pasture and animal productivity.

No adverse effects on well-being was found: Sherren et al. [66] published a study based on an online survey of 200 Canadian beef producers. The study reported high levels of physical well-being, systems thinking, non-traditional values, prioritizing enjoyment of life, and a tendency to use a wide range of methods to learn about adaptive multi-paddock (AMP) grazing among ranchers. Similarly, Vivas and Hodbod [67] concluded that the wider adoption of regenerative grazing effectively operationalizes social well-being.

### 8.5. Positive Ecological Impact

van Eekeren et al. [68] have concluded that a transition towards regenerative agricultural systems is supposed to enhance functional agro-biodiversity and create more resilient systems. For beef farming, the extensification towards semi-natural grasslands and establishing multi-species grasslands are potential contributors to this transition. Both grassland types differ in terms of plant diversity and management, but it is unclear whether and how these differences translate into soil quality and soil biodiversity.

### 8.6. Reduce Beef-Sector Emissions

Some works of research demonstrate that mitigation practices can substantially reduce beef-sector emissions. Chen et al. [2] identify the improved feed digestibility, enhanced manure management, and increased utilization of crop residues as effective measures. Pelton et al. [5] similarly report that combining 42 practices—including feed additives, cover cropping, and energy efficiency interventions—could cut US beef emissions by up to 30%. These studies collectively highlight the potential for integrated, system-level approaches to balance the productivity, sustainability, and regenerative system.

### 8.7. Long-Term Impact

According to Messner et al. [57], the insights from the research are significant given the growing evidence for the benefits of a regenerative form of management covering vast areas of lands during an area characterized by the pressures of climate change and the need to sequester carbon and support the conservation and regeneration of natural systems.

## 9. Regenerative Development Opportunity

The winners of the 2025 Nobel Prize in Economics (Joel Mokyr, Philippe Aghion, and Peter Howitt) concluded that ‘sustained economic growth, a modern phenomenon, is driven by technology, innovation and “creative destruction”. However, this requires an open society, interaction between science and technology, and policies that foster competition while supporting those displaced by change’ [69,70,71,72,73].

Although the researchers’ analysis is economics-based, the aforementioned statement may also be valid for the regenerative development of beef cattle farming from many perspectives. In our opinion, based on the study of the cited publications, the current state of science and technology in this field suggests the following possibilities for regenerative beef cattle farming:

- Grazing-based beef systems offer significant potential for innovations aimed at improving productivity, sustainability, animal welfare, and resilience. One major opportunity lies in precision livestock technologies, such as GPS collars, virtual fencing, and remote sensing tools, which allow farmers to monitor cattle movement, grazing intensity, and pasture health in real time. These tools help to optimize rotational grazing, prevent overgrazing, and reduce labor requirements.
- Another area of innovation is regenerative pasture management. This includes multi-species swards, adaptive multi-paddock grazing, enhancing soil carbon, and biodiversity-friendly grazing regimes. These methods can improve the quality of forage, increase the resilience to drought, enhance soil fertility, and reduce the dependency on external resources.
- There is also growing potential in breeding and genetic selection that is specifically tailored to grazing systems. Selecting traits such as pasture feed efficiency, heat tolerance, parasite resistance, and maternal performance can boost herd productivity while reducing costs.
- Innovations in animal health and welfare, such as non-antibiotic parasite control, automated health monitoring, and stress reduction through improved grazing design, further support more robust and profitable systems.
- Finally, market and value-chain innovations such as carbon credits, payments for ecosystem services, traceability systems, and branded, high-quality grass-fed beef create new income streams for producers engaged in sustainable grazing practices.

In summary, the focus of innovation in grazing-based beef production is on integrating precision technologies, regenerative grazing methods, genetic improvements, and new market opportunities to create livestock systems that are more productive, resilient, and environmentally beneficial. Regenerative cattle are framed as ancestral keystone species and prospective ecological engineers, concepts associated with distinct meanings and biopolitical consequences mentioned by Cusworth et al. [73]. As keystone species, they are attributed to ecological agency in driving ecological restoration, biodiversity, and ecosystem functions at a landscape scale. As ecological engineers, they are positioned within planetary greenhouse gas regulation through carbon sequestration and methane emissions reductions.

Regenerative beef cattle production must simultaneously promote the sustainability of pasture ecosystems and ensure economic resilience under conditions of fluctuating market prices for cattle, feed, and other production inputs. The development and implementation of management strategies capable of achieving production objectives while preserving soil quality and ecosystem integrity, as well as minimizing environmental impacts, require a comprehensive understanding of how grazing intensity, stocking rate, and grazing duration affect cattle performance. In addition, it is essential to determine how regenerative systems contribute to the maintenance of productive and ecologically stable pasture resources, and how innovations in feeding and watering infrastructure can mitigate adverse impacts on soil and water quality. Furthermore, supporting nutrient cycling, reducing the environmental impacts, and optimizing the input costs are also of critical importance.

## 10. Conclusions

From the early hunter-gatherers who lived in harmony with nature, to modern societies grappling with global ecological crises, humanity’s story is intricately linked to the exploitation of natural resources. While our ancestors had a light impact on the planet, modern humans consume far more than the Earth can sustain. The future of humanity depends on us learning from the past and using our knowledge and technology to protect and restore the natural world that sustains us, rather than exploit it.

Although beef production has a much smaller environmental impact than urbanization and other sectors of agriculture, beef farmers still need to consider regenerative development for the future.

While the cow–calf phase is responsible for the largest share of the beef sector’s GHG footprint, it also represents the greatest opportunity for regenerative transformation. By adopting practices such as adaptive grazing, forage diversity, and soil health management, ranchers can transition from emission-intensive systems to those that are carbon-neutral or even carbon-negative, thereby contributing to both climate mitigation and ecosystem restoration [42,43].

Based on experiments, the concept of regenerative beef farming appears to be viable. It is based on three main dimensions: animal welfare, improved biodiversity, and human health. Moderate intensification can promote innovation, reduce the area occupied by livestock, i.e., beef cattle farming, and mitigate its socio-environmental impacts, promoting a more sustainable model of beef production [69].

Regenerative-grazing-based beef cattle farming shifts the goal of pasture management from maximizing short-term livestock output to restoring ecological processes, creating a sustainable balance between production and environmental health. However, regenerative livestock, regenerative cow-calf production per se, as an emerging paradigm is very promising but still requires more local research [70].

Scientific innovation in grazing-based beef cattle systems must focus on precision livestock monitoring, regenerative pasture management, genetic selection, animal health improvements, and opportunities within the value chain. These innovations help optimize productivity, enhance sustainability, improve animal welfare, and create new income streams through ecosystem services or premium grass-fed beef markets.

However, regenerative cattle farming has many advantages, and it is important to draw attention to some risk factors. The transition to regenerative practice is not easy and additional costs may arise. Since not all farmers are equipped for this, technical assistance and producer training would be necessary. There may also be economic barriers, and there is a lack of certifications and reliable ways to measure regenerative outcomes.

Before regenerative agricultural practices are communicated to consumers or the broader public, a more robust body of knowledge is required at the cattle producer level. Further research is necessary to address the existing gaps in both practical knowledge and scientific understanding. The effective knowledge dissemination of these practices is essential. The adoption of regenerative agriculture requires sustained commitment, patience, and long-term strategic thinking, as meaningful progress is likely to occur only over an extended period.

We believe that our work is novel in drawing attention to the need for, and importance of, regenerative development. In our opinion, this could represent a paradigm shift. Previously, the emphasis was on sustainable development. Regenerative development encompasses much more than sustainability. The latter covers not only the economic aspects, but also environmental protection and nature conservation. We believe that this perspective will become increasingly important in the future, not only in beef cattle farming, but also in other areas of agriculture.

We recommend that forage and beef cattle researchers expand and improve the dissemination of research findings related to these systems through social media content that is targeted, concise, free from scientific jargon, and capable of being carried out by farmers.

Regenerative systems should be regarded as systems under continuous development, because management practices continually refine strategies and implementation systems in order to ensure sustainable pasture-based beef cattle production, ecosystem services beneficial to soil and water components, and favorable economic returns.

## Data Availability

No new data were created or analyzed in this study. Data sharing is not applicable to this article.
